# Association of Galectin 9 Expression with Immune Cell Infiltration, Programmed Cell Death Ligand-1 Expression, and Patient’s Clinical Outcome in Triple-Negative Breast Cancer

**DOI:** 10.3390/biomedicines9101383

**Published:** 2021-10-03

**Authors:** Mi-Ha Ju, Kyung-Do Byun, Eun-Hwa Park, Jin-Hwa Lee, Song-Hee Han

**Affiliations:** 1 Department of Pathology, Dong-A University College of Medicine, Busan 49201, Korea; miha337@nate.com; 2 Department of Surgery, Dong-A University Hospital, Dong-A University College of Medicine, Busan 49201, Korea; kdbyun@dau.ac.kr (K.-D.B.); parkeh@dau.ac.kr (E.-H.P.); 3 Department of Radiology, Dong-A University Hospital, Dong-A University College of Medicine, Busan 49201, Korea; jhrad@dau.ac.kr

**Keywords:** Galectin-9, triple-negative breast cancer, tumor-infiltrating lymphocyte, PD-L1

## Abstract

Galectin-9 (Gal-9) is an immune checkpoint protein that facilitates T cell exhaustion and modulates the tumor-associated microenvironment, and could be a potential target for immune checkpoint inhibition. This study was conducted to assess Gal-9 expression in triple-negative breast cancer (TNBC) and evaluate its association with programmed cell death ligand 1 (PD-L1) expression and immune cell infiltration in tumors and the clinical outcome of patients. Overall, 109 patients with TNBC were included. Gal-9 expression was assessed its relationships with tumor clinicopathologic characteristics, tumor-infiltrating lymphocyte (TIL) levels, PD-L1+ immune cells, and tumor cells by tissue microarray and immunohistochemistry. Low Gal-9 expression was statistically correlated with higher tumor stage (p = 0.031) and presence of lymphovascular invasion (*p* = 0.008). High Gal-9 expression was associated with a high level of stromal TILs (sTIL; *p* = 0.011) and positive PD-L1 expression on tumor cells (*p* = 0.004). In survival analyses, low Gal-9 expression was associated with significantly poor OS (*p* = 0.013) in patients with TNBC with PD-L1 negativity in tumor cells. Our findings suggest that increased Gal-9 expression is associated with changes in the antitumor microenvironment, such as increased immune cell infiltration and antimetastatic changes. This study emphasizes the predictive value and promising clinical applications of Gal-9 in TNBC.

## 1. Introduction

The immune system is a vital part of the tumor environment. After the recognition of the epidemiological association between the immune response and cancer, the idea of utilizing the immune system as a means to eliminate malignant cells arose [1]. The concept of immunotherapy against cancer cells has gained much support with new technology and scientific experiments. A study with mice lacking interferon-γ (IFN-γ) demonstrated the rapid growth of tumors in the mice [2]. The advent of genetically deficient mice in a crucial immune system component has demonstrated a link between cancer immunosurveillance and cancer development [3,4]. The fact that the immune system has played a leading role in preventing carcinogenesis is now fundamentally recognized in oncology.

In solid tumors, the tumor microenvironment includes the extracellular matrix and various cellular components, such as stromal, endothelial, and immune cells. Tumor-infiltrating lymphocytes (TIL) consist of all populations of lymphocytes that have invaded the intratumoral and intertumoral areas. Some tumors are profoundly infiltrated by immune cells, while in others, only subtle infiltration is detectable. These TILs are emerging as markers to assess immune reactivity and important biomarkers for predicting favorable prognosis in cancers, including breast cancer [5].

Triple-negative breast cancers (TNBCs) represent 15–20% of breast carcinomas and are defined by the lack of expression of estrogen receptor (ER) and progesterone receptor (PR) and the lack of overexpression and/or amplification of human epidermal growth factor receptor 2 (HER2). Patients with TNBC do not receive endocrine or HER2-targeted therapy and have limited targeted therapeutic options. Furthermore, TNBCs are highly proliferative tumors and have an unfavorable prognosis and aggressive biology, including rapid onset of metastasis and recurrence after adjuvant chemotherapy [6]. In the immunology field, recent advances in genomics have underlined the immunogenicity of TNBC, although breast cancer has traditionally been regarded as a poorly immunogenic, cold tumor for a long time. TNBC has the highest mutation frequency of breast cancer subtypes [7,8], which leads to an increased chance of immunogenic mutations generating neoantigens [9]. Thus, TNBCs are now thought to be the most immunogenic subtype. The presence of TIL has been reported to be positively correlated with more prolonged survival, particularly in TNBC [10]. Some researchers have demonstrated higher rates of programmed cell death ligand 1 (PD-L1) expression in TNBC patients than in patients with other breast cancers [11,12,13] and better survival with treatments that target PD1/PD-L1 in TNBC [14]. The Food and Drug Administration (FDA) recently approved the use of atezolizumab and nab-paclitaxel chemotherapy for the first-line treatment of patients with locally advanced and metastatic TNBC [15].

Targeting immune checkpoints, such as PD-L1, with immune checkpoint-inhibiting molecules has dramatically changed treatment paradigms in medical oncology. Currently, several of these types of interactions are being investigated. Gal-9 is an additional negative checkpoint of the immune response in the context of the tumor microenvironment [16]. Gal-9 has been reported to serve as a binding partner for T cell immunoglobulin mucin 3 (TIM-3), a receptor related to T cell exhaustion, and induces T cell apoptosis and suppresses antigen-specific cytotoxic T lymphocyte (CTL) effector functions [17]. In addition, Gal-9 restricts the differentiation and function of T helper 17 (Th17) cells and promotes the induction of regulatory T cells (Tregs) independently of TIM-3. Furthermore, Gal-9 was found to be simultaneously expressed with PD-1, and the presence of Gal-9 was associated with terminally exhausted T cells [18]. Many clinical studies have demonstrated that the expression of Gal-9 has a close association with metastasis and recurrence in solid tumors, including melanoma [19], gastric cancer [20], hepatocellular cancer [21], lung cancer [22], and breast cancer [23]. However, conflicting studies exist regarding changes in the antitumor environment. Some previous studies reported that loss of Gal-9 expression was identified during the course of tumorigenesis [24]. Though there is a promising microenvironment-modulatory ability of Gal-9, the consistency and magnitude of the prognostic impact of GAL-9 remain controversial in TNBC.

Thus, this study aimed to examine GAL-9 expression in TNBC and its relationship with TIL, the additional immune inhibitory molecule PD-L1 expression, and cancer-related survival.

## 2. Materials and Methods

### 2.1. Selection of TNBC Patients and Tissue Microarray Construction

The study population consisted of 109 TNBC patients (with ER-negative, PR-negative, and HER2-negative disease) who were treated at Dong-A University Hospital (Busan, Korea) between 2007 and 2011. None of the patients received any neoadjuvant chemotherapy or radiation before surgery. Surgical pathologic reports and clinical information, including sex, age, and stage, were reviewed. Breast cancer stage was recategorized based on the eighth edition of the Cancer Staging Manual of the American Joint Committee on Cancer. The clinicopathological information of the TNBC patients is given in Table 1. The clinical study protocol was approved by the Institutional Review Board at the Dong-A University Hospital, Busan, Republic of Korea (DAUHIRB-21-040). The requirement for obtaining informed consent from patients was waived due to the use of archived formalin-fixed and paraffin-embedded (FFPE) tumor tissues.

We prepared a tissue microarray (TMA) with 109 TNBC cancer tissue punches from FFPE tumor samples according to a previously described process [25]. Numerous cancer cell areas with high levels of TIL on hematoxylin and eosin-stained slides were identified, and two 3-mm tissue cores from individual tumors were obtained. TMAs were constructed with a tissue arrayer (Unitma Co., Ltd., Seoul, Korea). Ten TMA blocks were constructed.

### 2.2. Evaluation of Stromal Tumor-Infiltrating Lymphocytes

Surgical specimens were fixed in buffered formalin solution, cut into 4 µm-thick slices, and stained with hematoxylin and eosin. Using an optical microscope at ×200 and ×400 magnification, a surgical pathologist quantified the level of stromal TIL (sTIL). This level classified into the following three grades per the International TILs Working Group (10) criteria: low (sTIL: <10%), intermediate (sTIL: ≥10 and ≤40%), and high (sTIL: >40%).

### 2.3. Immunohistochemistry and Scoring

Immunohistochemistry (IHC) staining for ER, PR, HER2, Ki-67, Gal-3, and PD-L1 was performed with a BenchMark XT automated immunostainer (Ventana Medical Systems Inc., Tucson, AZ, USA) using an Ultraview Universal DAB Detection Kit (Ventana Medical Systems Inc.). After deparaffinization, rehydration, and antigen retrieval, diluted primary anti-Gal-9 rabbit monoclonal antibodies (D9R4A; Cell Signaling Technology, Beverly, MA, USA. 1:100) and anti-PD-L1 rabbit monoclonal antibodies (E1L3N; Cell Signaling Technology; 1:100) were added and incubated. The expression of key biomarkers, including ER (1:50), PR (1:50), HER2 (1:100), and Ki-67 (1:800) were evaluated in surgical specimens at the time of diagnosis based on the American Society of Clinical Oncology (ASCO)/College of American Pathologists (CAP) guideline recommendations [26,27,28]. High Ki-67 proliferation index was defined as staining in 20% or more of the tumor cells [29]

**Figure 1:**
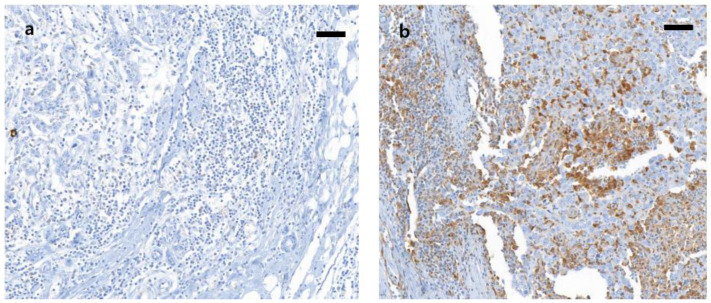
Representative cases were showing Gal-9 expression. (**a**) Low Gal-9 expression; (**b**) high Gal-9 expression (x200, respectively; scale bar, 100 µm).

For the evaluation of Gal-9, the intensity of staining and percentage of immune cells were evaluated. The signal strength of Gal-9 staining in normal luminal cells was used as a reference point for determining the expression intensity [30]. The intensity of expression was classified into four categories: 0, negative; 1+, weakly positive; 2+, moderately positive, and 3+, strongly positive. Weakly positive (1+) was defined as staining of the cell cytoplasm or nuclei that was weaker than that of normal luminal cells. The immune cells that showed staining equivalent to that in the control were considered moderately positive, and those that showed staining that was stronger than that in the internal control were considered strongly positive. The percentage of stained cells (0 = 0%, 1 = 1–10%, 2 = 11–50%, 3 = 51–100%) was assessed and multiplied by the staining intensity value, resulting in the histologic score. For statistical analysis, high expression was defined as a score of 4 to 9, and low expression was defined as a score of 0 to 3 [31,32]. Representative staining is shown in Figure 1. For PD-L1, the percentage of PD-L1 expression in invasive tumor cells was calculated as the number of invasive carcinoma cells showing either partial or complete cell membrane staining of any intensity divided by the total number of invasive carcinoma cells. The percentage of PD-L1 expression in tumor-infiltrating immune cells was calculated as the proportion of the intratumoral or peritumoral stromal rim occupied by PD-L1-positive immune cells of any staining intensity. Positivity was defined as ≥1% in both tumor cells and immune cells [33]. All slides that were immunohistochemically stained were read by two experienced pathologists blinded to other clinical information.

### 2.4. Statistical Analysis

Statistical analysis was performed using SPSS software (version 22.0; SPSS, Inc.; Chicago, IL). The relationships of Gal-9 expression with clinicopathological characteristics were assessed using the chi-square test. Disease-free survival (DFS) and overall survival (OS) analyses based on Gal-9 expression were performed with the Kaplan–Meier method with the log-rank test. Univariate survival analysis with individual covariates and multivariate survival analyses were performed using the Cox proportional hazards regression model to assess whether the expression level of Gal-9 was an independent predictor of disease relapse or survival in TNBC. In all the tests, statistical significance was defined as *p* < 0.05.

## 3. Results

### 3.1. Clinicopathological Characteristics

There were 109 patients in total. The clinicopathological features are summarized in Table 1. The median age of the patients was 50 years (range 30–74 years). All patients were women. In the cohort as a whole, just over half of the patients had pathologic tumor stage 2–3 disease (62, 56.8%) (Table 2). Most patients (108, 99.0%) received treatment after surgery, including chemotherapy or radiation therapy, while 82 patients (75.2%) underwent both chemotherapy and radiation therapy. No patients received neoadjuvant treatment.

### 3.2. Galectin-9 Expression and its Correlation with Clinical and Pathologic Characteristics and Immune Status

Of the specimens, 22 (20.1%) specimens of TNBC had low Gal-9 expression, and 87 (79.9%) had high Gal-9 expression. Pathologically, low Gal-9 expression was associated with higher tumor stage (*p* = 0.031) and presence of lymphovascular invasion (*p* = 0.008) than high Gal-9 expression. High expression of Gal-9 also tended to be related to a high Ki-67 labeling index (*p* = 0.067)

### 3.3. Correlation between Galectin-9 Expression and PD-L1 Expression and sTIL Level

First, we examined the correlation of Gal-9 expression with the expression of immune checkpoint molecules such as PD-L1 and the level of sTIL. We detected that high Gal-9 expression was robustly associated with the immune status of TNBC. High Gal-9 expression was associated with a high level of sTIL (*p* = 0.011), positive PD-L1 expression on tumor cells (*p* = 0.004), and negative PD-L1 expression on immune cells (*p* = 0.001) (Table 3).

### 3.4. Prognostic Significance of Galectin-9 Expression and its Association with Immune Status in TNBC

**Figure 2:**
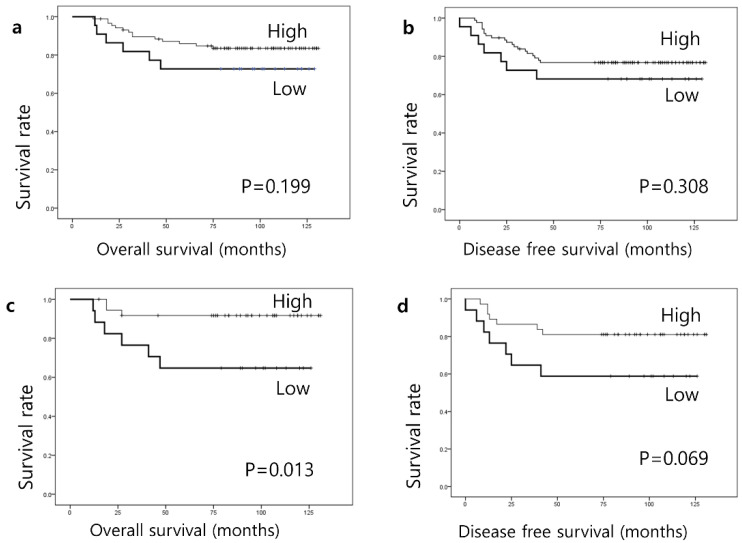
Kaplan–Meier survival analysis according to the Galectin-9 expression level in the overall cohort (**a**,**b**) and cases with negative PD-L1 expression (**c**,**d**).

Finally, we evaluated the clinical prognosis of patients with TNBC. The median follow-up time was 76 months (range, 6–131 months). DFS was defined as the duration from the date of initial diagnosis to the first detection of breast cancer-specific relapse or death. OS was defined as the time interval from the date of initial diagnosis to the date of breast cancer-related death. Kaplan–Meier analysis revealed that the expression level of Gal-9 was not associated with OS (*p* = 0.199) or DFS (*p* = 0.308) in TNBC (Figure 2). Furthermore, the level of sTIL and the expression of PD-L1 in immune cells or tumor cells did not show a statistically significant association with OS or DFS. Interestingly, low expression of Gal-9 was associated with significantly shorter OS (*p* = 0.013) and tended to be associated with poor DFS (*p* = 0.069) in patients with TNBC with PD-L1 negativity in tumor cells (Figure 2). However, in patients with PD-L1 positivity, no significant results were obtained. In the multivariate analysis, pathologic tumor stage (*p* = 0.024) and lymph node stage (*p* = 0.011), but not Gal-9 expression, were independent prognostic factors for the DFS of patients with TNBC (Table 4). Pathological tumor stage (*p* = 0.004) and lymph node stage (*p* = 0.001) were associated with OS. Patients were stratified based on PD-L1 expression on tumor cells and immune cells, but no significant differences in OS and DFS were found.

## 4. Discussion

Along with tissue invasion, angiogenesis, and local tissue necrosis and hypoxia, evasion of the antitumor immune response also promotes breast cancer pathogenesis [34]. Thus, tumor-associated immunosuppression has attracted notable interest from researchers. Immunological checkpoint inhibitors have shown encouraging results in some malignancies, prompting cancer researchers to propose that checkpoint inhibitors can achieve the same outcome in breast cancer. In addition to developing a variety of checkpoint inhibitors, related clinical trials are actively ongoing, providing therapeutic strategies and hope for TNBC patients [35].

Gal-9 is part of a family of β-galactoside-binding lectins and has tandem repeats with two separate carbohydrate-recognition domains (CRDs) joined by a short polypeptide called the linker domain [36]. Functionally, the binding of galectin to its ligands was shown to mediate cell–cell and cell–pathogen interactions in the tumor-associated microenvironment, including immunity and microenvironment modulation. Several studies have been published regarding the role of Gal-9 in immune regulation. In a study by Zhang et al. [37], Gal-9 promoted the induction of myeloid-derived suppressor cells in their microenvironment, leading to myeloid cell-mediated T cell inhibition. A previous study conducted by Sehrawat et al. [38] found that Gal-9 contributes to inducing T cell exhaustion. T cell exhaustion is characterized by the stepwise and progressive loss of effective functions such as cytotoxicity and proliferation and results in impaired proliferation and decreased production of effector molecules in response to tumor antigens. In addition, gal-9 promoted the activity of Forkhead box P3-positive Tregs and hindered the immune response of effector T lymphocytes with TIM-3 [36]. This evidence suggests that Gal-9 contributes to mediating immunosuppressive functions. However, Gal-9 also acts as a proinflammatory agent. According to Matsuura et al. [39], Gal-9 was responsive to lipopolysaccharide. It could form a compound with nuclear factor (NF)-IL6 to transactivate several proinflammatory cytokines, including IL-1α, IL-1β, and IFN-γ, in monocytes [39]. Gal-9 also induced maturation of dendritic cells, promoting Th1 immune responses in a concentration-dependent manner [40]. This experimental evidence revealed that Gal-9 has diverse immunomodulatory influences depending on its concentration and interaction partners. Considering its significant function in immunology, investigating the effect of Gal-9 on TIL of TNBC is warranted.

In the present study, we explored the status of Gal-9 expression in TNBC. We observed a significant positive correlation between Gal-9 expression and the level of sTIL. Furthermore, we discovered that high expression of Gal-9 was associated with a higher TIL degree and the presence of tumoral PD-L1 expression. Patient clinical factors, such as lymphovascular invasion and higher pathologic tumor stage, were related to low Gal-9 expression. 

As mentioned earlier, Gal-9 also plays a pivotal role in the tumor microenvironment. The tumor microenvironment comprises multiple cell types, including fibroblasts, adipose cells, immune-inflammatory cells, and blood and lymphatic vascular networks. Gal-9 is involved in cell-matrix interactions and appears to regulate adhesion at multiple levels depending on the cancer type, both directly and indirectly. A study by Irie et al. [41] demonstrated that cytoplasmic Gal-9 induces cancer cell aggregation, leading to inhibition of metastasis. This phenomenon has been observed in melanoma [19], hepatocellular carcinoma [42], and breast cancer [41] cells. In such studies, cytoplasmic Gal-9 was observed to be associated with the aggregation of cells, which inhibited cell invasion, detachment from the tumor, and attachment to the vascular endothelium. Not only these findings but also the immunogenic effects that we mentioned earlier explain the mechanism underlying the behavior of Gal-9 in our study.

TIL are deemed responsible for the host immune response against cancer. Several studies have shown that a high level of sTIL is more common and predictive of a positive long-term prognosis in TNBC than in other types of breast cancer [5,43]. However, the prognostic effect of PD-L1 expression on both neoplastic and inflammatory cells has been controversial. A study reported by Mori et al. [44] showed a significant association between PD-L1 expression on tumor cells and the percentage of sTIL in surgical breast specimens and demonstrated that the interaction between TIL and PD-L1 led to a better clinical outcome. Antoni et al. [45] reported that the presence of T cells, which have the T-cell receptors to distinguish analogous tumor antigen on cancer cells, as well as the expression of PD-L1, is essential to increase treatment response to Anti-PD-L1 therapy. In this study, we observed that a higher level of sTIL and tumoral PD-L1 expression were associated with a high level of Gal-9 expression. Thus it is thought that the Gal-9 expression level may be additionally used to predict immune conditions, such as PD-L1 regulation or sTIL degree in patients with PD-L1 positive expression. Nevertheless, more large-scale studies need to be conducted to confirm the idea above.

To date, a few studies have explored the prognostic role of Gal-9 in various malignancies. Knudsen et al. [46] observed increased Gal-9 expression in glioblastoma, but its expression showed no prognostic value. In colon cancer, Wang et al. [47] reported that OS was longer in patients with high Gal-9 expression, presumably due to Gal-9 promoting the recruitment of natural killer (NK) cells. A study of breast cancer by Irie confirmed the significant correlation of positive Gal-9 expression with longer OS for patients with breast cancer and the status of distant metastasis [41]. In our study, there was no significant association between Gal-9 expression and OS or DFS. 

Expression of PD-L1 in tumor cells indicates the evasion of the T cell response [48]. In other words, the tumor cells with negative PD-L1 have the disadvantage of immunosurveillance and should escape through another checkpoint or immune escape mechanism. Interestingly, low GAL-9 expression in patients with negative PD-L1 expression had a statistically significant relationship with shorter OS in our research. Regretfully, few investigations were carried out to explore the mechanisms behind the correlation between Gal-9 expression and other immune escape mechanisms to date. However, considering the broad spectrum of immunomodulatory roles of Gal-9 in various cells, we carefully hypothesize that the tumors with negative PD-L1 regulation may be associated with Gal-9 associated reactions. However, the impact of such interactions on developing strategies to escape immune surveillance requires further investigations. There are some limited data: the heterogeneity of cancer subtype and the subpopulation of TIL. TNBC is a highly heterogeneous group of cancers. Lehmann et al. [49] initially defined six molecular subtypes by genomic and transcriptomic profiling: basal-like, mesenchymal, mesenchymal stem-like, immunomodulatory, and luminal androgen receptor, as well as an unspecified group. Furthermore, not only the degree of TILs but also the profile of TIL is crucial in the tumor microenvironment. Therefore, it is necessary to conduct more studies to clarify the actual role of Gal-9 concentrated on the molecular features of TNBC and the profile of TIL.

## 5. Conclusions

In conclusion, we showed that Gal-9 can predict tumor microenvironment characteristicsAlthough additional research into the underlying mechanisms and immune cell subset analysis is necessary, Gal-9 expression combined with PD-L1 expression may be helpful for stratification of TNBC patients and for predicting their prognosis. Our findings support the development of novel immune-targeted therapies, including agents such as PD-1/PD-L1 inhibitors and other targeted therapies for patients with TNBC.

## Figures and Tables

**Table 1:** Patient and tumor characteristics of the study cohort.

Clinicopathologic Characteristics	Number (%)
Age (years)	
Median (range)	50 (30–74)
T stage	
T1	47 (40.5)
T2	55 (50.5)
T3	7 (6.4)
N stage	
N0	74 (67.9)
N1	16 (14.7)
N2	6 (5.5)
N3	13 (11.9)
Histologic grade	
1	4 (3.7)
2	21 (19.3)
3	84 (77.1)
EIC	
Absent	96 (88.1)
Present	13 (11.9)
Lymphovascular invasion	
Absent	83 (76.1)
Present	26 (23.9)
Ki-67 labeling index	
<20%	28 (25.7)
≥20%	81 (74.3)
Stromal TIL percentage	
0–10%	18 (16.5)
11–40%	44 (40.4)
≥40%	47 (43.1)
PD-L1 expression in tumor cells	
<1%	54 (49.5)
≥1%	55 (50.5)
PD-L1 expression in lymphocytes	
<1%	67 (61.5)
≥1%	42 (38.5)
P53 overexpression	
Absent	46 (42.2)
Present	63 (57.8)
Adjuvant therapy	
Chemotherapy	107 (99.1)
Radiation	82 (75.2)

**Table 2:** Clinicopathologic characteristics according to Galectin-9 expression level.

Variable	Gal-9 Expression		*p* Value
	Low	High	
Age			
<50 years	11 (50%)	42 (48.3)	0.885
≥50 years	11 (50%)	45 (51.7)	
pT stage			
T1	5 (22.7)	42 (48.3)	0.031
T2–3	17 (77.3)	45 (51.7)	
pN stage			
N0	13 (59.1)	61 (70.1)	0.322
N1–3	9 (40.9)	26 (29.9)	
Histologic grade			
1–2	6 (27.3)	19 (21.8)	0.588
3	16 (72.7)	68 (78.2)	
Ki-67 labeling index			0.067
<20%	9 (40.9)	19 (21.8)	
≥20%	13 (59.1)	68 (78.2)	
Extensive intraductal component			0.081
Absent	17 (77.3)	79 (90.8)	
Present	5 (22.7)	8 (9.2)	
Lymphovascular invasion			0.008
Absent	12 (54.5)	71 (81.6)	
Present	10 (45.5)	16 (18.4)	
P53 overexpression			0.270
Absent	7 (31.8)	39 (44.8)	
Present	15 (68.2)	48 (55.2)	

**Table 3:** Immune status-based analysis stratified by Galectin-9 expression.

Variable	Gal-9 Expression		*p* Value
	Low	High	
Level of sTIL			0.011
Low	7 (31.8)	11 (12.6)	
Intermediate	11 (50.0)	33 (37.9)	
High	4 (18.2)	43 (49.4)	
PD-L1 expression in lymphocytes			0.001
<1%	20 (90.9)	47 (54.0)	
≥1%	2 (9.1)	40 (46.0)	
PD-L1 expression in tumor cells			0.004
<1%	17 (77.3)	37 (42.5)	
≥1%	5 (22.7)	50 (57.5)	

**Table 4:** Univariate and multivariate Cox regression analyses of patients with TNBC.

**Disease-** **F** **ree Survival**		**Univariate Analysis**		**Multivariate Analysis**	
**Variable**	**Category**	**HR (95% CI)**	***p* Value**	**HR (95% CI)**	***p* Value**
Age	≥50 years vs. <50 years	0.564–2.556	0.634		
T stage	T2-3 vs. T1	1.072–6.002	0.034	1.107–6.970	0.024
N stage	N1-N3 vs. N0	1.111–5.034	0.026	1.042–5.749	0.011
Histologic grade	III vs. I and II	0.442–2.716	0.842		
LVI	Present vs. absent	0.677–3.537	0.301		
sTIL level	High vs. low to intermediate	0.339–1.618	0.451		
PD-L1 expression in tumor cells	Positive vs. negative	0.410–1.854	0.721		
PD-L1 expression in lymphocytes	Positive vs. negative	0.502–2.330	0.842		
Ki-67 labeling index	High vs. low	1.055–10.610	0.041	1.055–10.410	0.047
Gal-9 expression	High vs. low	0.276–1.544	0.332		
**Overall Survival**		**Univariate Analysis**		**Multivariate Analysis**	
**Variable**	**Category**	**HR (95% CI)**	***p* Value**	**HR (95% CI)**	***p* Value**
Age	50 years vs. <50 years	0.486–2.602	0.784		
T stage	T2-3 vs. T1	2.071–37.944	0.003	2.144–37.279	0.004
N stage	N1-N3 vs. N0	1.551–8.508	0.003	1.783–9.503	0.001
Histologic grade	III vs. I and II	0.471–4.113	0.551		
LVI	Present vs. absent	0.711–4.285	0.224		
sTIL level	High vs. low to intermediate	0.702–4.224	0.235		
PD-L1 expression in tumor cells	Positive vs. negative	0.472–2.510	0.843		
PD-L1 expression in lymphocytes	Positive vs. negative	0.285–1.717	0.436		
Ki-67 labeling index	High vs. low	0.699–7.981	0.167	1.020–11.986	0.043
Gal-9 expression	High vs. low	0.241–1.577	0.313		
